# Additive effects of connectivity provided by different habitat types drive plant assembly

**DOI:** 10.1038/s41598-019-50184-2

**Published:** 2019-09-27

**Authors:** Léa Uroy, Cendrine Mony, Aude Ernoult

**Affiliations:** UMR CNRS ECOBIO, University of Rennes, Avenue du Général Leclerc, 35042 Rennes Cedex, France

**Keywords:** Biogeography, Community ecology

## Abstract

How connectivity affects plant assemblages is a central issue in landscape ecology. So far, empirical studies have produced contradictory results, possibly because studies: (1) inaccurately assess connectivity by prioritizing the respective effect of the type of habitat on plant assemblages and (2) omit the range of possible plant responses to connectivity depending on dispersal vectors. We focused on three dominant habitat types in agricultural landscapes (woodland, grassland and cropland), and analysed the effect of connectivity on herbaceous plant assemblage similarity for three primary dispersal modes (animal-dispersed, wind-dispersed and unassisted). Using circuit theory, we measured connectivity provided by woodland, grassland and cropland habitats independently. The similarity of plant assemblages was evaluated relative to the random expectation based on the regional pool. Overall, plant assemblage similarity in woodlands and temporary grasslands was dependent on connectivity, but not in wheat croplands. Only animal-dispersed species responded to connectivity. The similarity of animal-dispersed assemblages in woodlands was increased by the connectivity provided by woodland habitats, but was reduced by cropland habitats, whereas in temporary grasslands, similarity was increased by the connectivity provided by cropland habitats. Our results suggest that animal-dispersed species supplement their dispersal pathways, thus improving our knowledge of plant assembly rules in fragmented landscapes.

## Introduction

Habitat isolation is a major cause of loss of plant diversity in agricultural landscapes^1,2^. In such heterogeneous landscapes, plant species are distributed across a set of local communities linked through dispersal, which ensures their persistence as a metacommunity in the landscape^3,4^. Dispersal between communities is enhanced by landscape connectivity^5^. High connectivity enhances plant dispersal and increases genetic fluxes among local populations, thereby reducing the adverse effects of isolation. Therefore, high connectivity should enhance plant coexistence in multi-species assemblages. In experimental landscapes, connected patches have been shown to have higher species richness than unconnected patches^6^. This pattern is less clear in empirical studies where the relationship between connectivity and species richness and/or similarity in composition has been shown to be positive^7–9^, negative^10^, or absent^11,12^.

Inaccurate assessments of landscape connectivity may explain why empirical studies fail to demonstrate a common pattern. Assessing connectivity *in situ* is difficult and different approaches have been attempted. Some authors estimated connectivity through a binary landscape representation^13,14^ using the abundance and spatial configuration of patches of a given habitat (i.e. structural connectivity^15^). For example, the connectivity of a woodland fragment is calculated as a combination of the sizes of nearby woodland fragments and the Euclidean distance to those fragments [incidence function model index (IFM)^16,17^, e.g. Piessens *et al*.^7^, Lindborg & Eriksson^11^, Cousins *et al*.^12^]. Other studies improved these estimates of connectivity by using functional indices [e.g. integral index of connectivity (IIC)^18^ and probability of connectivity (PC)^19^]. Both IIC and PC indices incorporate dispersal and plant habitat specificities in addition to the abundance and spatial configuration of patches (i.e. potential functional connectivity^15^) and are based on graph theory (least-cost path distance^20^) or circuit theory (resistance distance^21,22^). Initially developed for animals, these indices are now increasingly used for plants although not yet widely at the plant community scale (but see Thiele *et al*.^8,9,23^ and Mony *et al*.^24^). These functional indices still consider that species which co-exist in plant communities may occupy different ecological niches and rely on different habitats to disperse^25^. Functional landscape connectivity is indeed calculated using resistance maps. These maps include all the landscape patches with associated resistance values per habitat type (i.e. the higher the type of habitat suitable to plant dispersal, the lower the habitat type resistance value), hence including the heterogeneity of the landscape matrix^13,26,27^. However, one limitation to using such indices is assigning resistance values, which is based on expert knowledge (i.e. subjective)^28–31^ and assumes a role hierarchy exerted by the various types of habitat on the connectivity among habitat patches. Understanding how the different habitat types between patches are independently involved in connectivity may thus improve our understanding of their effects on plant biodiversity. For example, a patch of a given habitat type can be connected to other patches of the same type by woodland, grassland and cropland habitat patches, even though their respective effect may differ. Improved understanding of the influence of landscape connectivity on species assemblages can be achieved by evaluating the additive influences of each type of habitat (the resulting effect, computed as the sum of the independent individual effect of the connectivity provided by each habitat type) on plant composition.

Considering each habitat type separately when assessing landscape connectivity is even more important in the case of plants. Plant seeds are dispersed in a landscape by a wide variety of vectors, whose response to connectivity is likely to differ depending on the type of habitat. In the case of animal-dispersed plant species, there is a broad consensus that animal movements are promoted by landscape connectivity in a wide range of habitats^6,32–35^. Therefore, all habitat types should facilitate dispersal of plants by animal species (e.g. by forest birds and mammals through woodland habitats^36–38^ and by amphibians through aquatic habitats^39^). However, for other modes of dispersal, plant dispersal is likely to depend on the type of habitat on which connectivity was based. For example, wind dispersal depends on prevailing winds. Open habitats (e.g. grasslands, grassy strips and croplands) affect wind dynamics (redirection and promotion of airflow) thereby increasing the likelihood of wind-dispersed seed uplift. In contrast, closed habitats (e.g. forests, woodlands and hedgerows) are known to impede wind dispersal^40–42^. Therefore, connectivity provided by open habitat patches should promote the dispersal of wind-dispersed species, while that provided by woodland habitats should impede their dispersal. In unassisted species (i.e. plant that are not dispersed by vectors), dispersal occurs step-by-step from the parent plant over successive generations because their seeds are not adapted for dispersal^43^. Because annual dispersal occurs over such short distances in unassisted species and because their progenies require a similar habitat to that of their parents, dispersal should be enhanced only by the connectivity provided by the focal habitat (habitat of interest) and inhibited by others. Accounting for the influence of connectivity provided by each habitat type according to the dispersal mode of plant species may provide insight into plant community assembly.

The aim of this study was to test the effect of landscape connectivity on herbaceous plant assemblages according to their dispersal mode. Our analysis focused on three habitat types typical of agricultural landscapes: woodland, grassland, and cropland. In these three habitat types, we assessed the response of plant assemblages to connectivity. Connectivity was measured as the addition of the independent effects of connectivity provided by the patches of each habitat type.

We calculated connectivity using the resistance distance for each habitat type. For a given habitat type, the effect of connectivity on plant assemblage similarity was tested with respect to each mode of dispersal (animal-dispersed, wind-dispersed and unassisted dispersal). Plant response was evaluated relative to the random similarity expected based on the regional species pool. In the three habitats, we specifically tested the following assumptions:

1. Similarity between plant assemblage pairs for a given focal habitat is better predicted by simultaneously accounting for the contribution of the various habitat types to connectivity rather than by only examining only the focal habitat type.
2. The independent effect of the connectivity provided by the different habitat types depends on the dispersal mode considered.
  - The similarity among animal-dispersed species assemblages is increased by connectivity provided by any habitat type.
  - The similarity among wind-dispersed species assemblages is increased by connectivity provided by open habitats (e.g. grassland and cropland) and reduced by closed habitats (e.g. woodland), because of their barrier effect
  - Due to the short dispersal distances of unassisted species, the similarity among such species assemblages is increased by connectivity provided by habitats of the same type and is reduced by others.

## Results

### Characteristics of the plant assemblages studied

**Figure 1:**
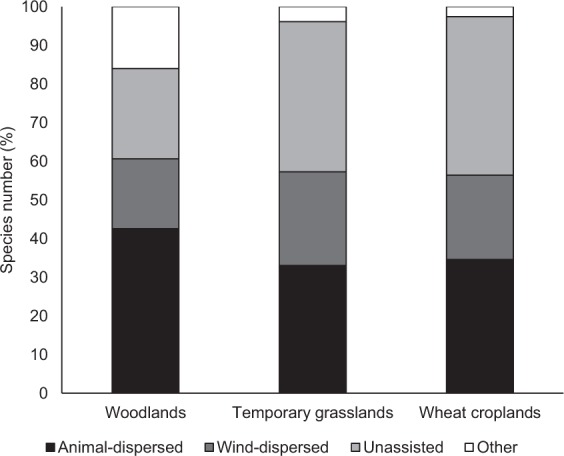
Number of herbaceous species (in percentage), by mode of dispersal and habitat type (woodland, temporary grassland, and wheat cropland). “Other” refers to water-dispersed species that were not included in this study.

The regional species pools of woodlands, temporary grasslands and wheat croplands contained 94, 103 and 78 herbaceous species, respectively. Similarity values between species pools using Sørensen indices were: 0.26 between woodlands and temporary grasslands, 0.23 between woodlands and wheat croplands and 0.53 between temporary grasslands and wheat croplands, revealing marked differences between plant assemblages in the three habitat types. Total herbaceous species richness according to habitat type and mode of dispersal were as follows: (1) woodland: 40 animal-dispersed, 34 wind-dispersed and 27 unassisted species, (2) temporary grassland: 17 animal-dispersed, 25 wind-dispersed and 17 unassisted species and (3) wheat cropland: 22 animal-dispersed, 40 wind-dispersed and 32 unassisted species. No exotic species were recorded in plant assemblages in woodlands, temporary grasslands or wheat croplands, based on the *List of invasive vascular plants in Brittany*^44^. Characteristics of the plant assemblages for woodlands, temporary grasslands and wheat croplands are summarized in Table 1 and Fig. 1.

**Table 1:** Characteristics of herbaceous species assemblages (species richness and Sørensen similarity index) for woodland, grassland and cropland habitats.

	Woodlands		Temporary grasslands		Wheat croplands	
	Mean (SD)	Min-Max	Mean (SD)	Min-Max	Mean (SD)	Min-Max
***Whole plant assemblages***						
Richness	15.8 (8.3)	2–36	26.9 (8.4)	17–51	13.6 (6.9)	3–36
Similarity	0.29 (0.15)	0–0.67	0.59 (0.07)	0.41–0.75	0.29 (0.14)	0–0.67
***Animal-dispersed assemblages***						
Richness	8.7 (3.6)	2–17	8.80 (2.9)	4–14	4.3 (2.2)	0–10
Similarity	0.37 (0.17)	0–0.80	0.62 (0.12)	0.35–0.92	0.22 (0.21)	0–0.89
***Wind-dispersed assemblages***						
Richness	1.6 (1.4)	0–5	6.6 (2.8)	3–15	2.5 (2.0)	0–8
Similarity	0.09 (0.20)	0–1	0.57 (0.14)	0.17–0.91	0.37 (0.17)	0–0.80
***Unassisted assemblages***						
Richness	3.7 (2.8)	0–12	11.1 (3.7)	6–22	6.6 (3.6)	1–19
Similarity	0.20 (0.22)	0–0.80	0.60 (0.11)	0.35–0.90	0.36 (0.17)	0–0.86

Plant assemblages were sampled 25 patches of woodlands [sampling area = 6 × (14 × 5 m) quadrats = 420 m²], temporary grasslands [sampling area = 10 × (5 × 5 m) quadrats = 250 m²] and wheat croplands [sampling area = 5 × (5 × 5 m) quadrats = 125 m²] selected for the study. SD = standard deviation.

### Effect of connectivity on assemblage similarity

**Figure 2:**
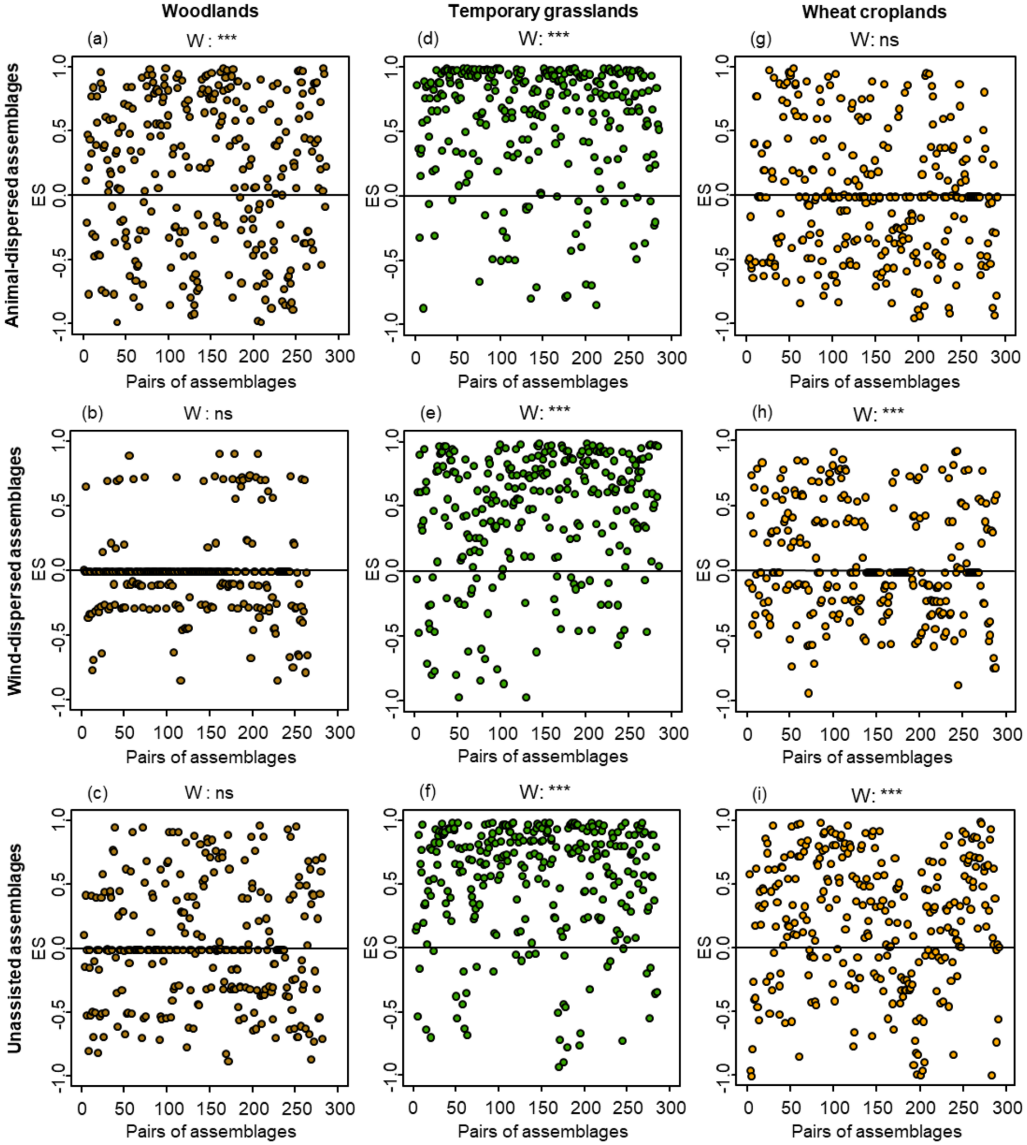
Effect size (ES) of similarity values for all pairs of animal-dispersed, wind-dispersed, and unassisted assemblages in habitats of woodland (brown dots), temporary grassland (green dots) and wheat cropland (yellow dots). P-values for the Wilcoxon tests (W) are on each graph. ***: p < 0.001; **: p < 0.01; *: p < 0.05; ns (not significant): p > 0.05.

We demonstrated a non-random pattern of plant similarity between patches for all assemblages, except for three models (Fig. 2): wind-dispersed and unassisted plant assemblages in woodlands and animal-dispersed plant assemblages in wheat croplands. For the non-random patterns, effect size (ES) of similarity values were best explained by models that included resistance distance (distR) of the various habitat types than by models that included only distR of the same habitat type (Supplementary Table S1). In most cases, the probability of the distR of the other habitat types to appear in the best model explaining ES values was at least equal to the probability of the distR of the same habitat type, as indicated by their relative importance (Table 2).

**Table 2:** Summary of the model-averaged estimates, relative importance (RI) and 95% confidence interval (CI) of the independent variable(s) according to the AICc framework.

	Models			Intercept	DistR woodlands			DistR grasslands			DistR croplands		
	N	R²m	R²c		Estimate	RI	CI	Estimate	RI	CI	Estimate	RI	CI
**Woodlands**													
*Animal-dispersed assemblages*													
ES	283	0.09	0.48	**0.20**	**−0.32**	**1.00**	**(−0.53, −0.10)**	0.06	0.44	(**−**0.12, 0.23)	**0.21**	**1.00**	**(0.07, 0.34)**
*Wind-dispersed assemblages*													
ES	—	—	—	—	—	—	—	—	—	—	—	—	—
*Unassisted assemblages*													
ES	—	—	—	—	—	—	—	—	—	—	—	—	—
**Temporary grasslands**													
*Animal-dispersed assemblages*													
ES	283	0.09	0.56	0.62	0.01	0.37	(**−**0.06, 0.09)	0.03	0.49	(**−**0.05, 0.10)	**−0.13**	**1.00**	**(−0.22, −0.04)**
*Wind-dispersed assemblages*													
ES	283	0.00	0.62	0.50	**−**0.01	0.32	(**−**0.06, 0.05)	**−**0.01	0.36	(**−**0.06, 0.04)	**−**0.00	0.26	(**−**0.05, 0.04)
*Unassisted assemblages*													
ES	283	0.04	0.45	0.59	**−**0.07	0.74	(**−**0.19, 0.05)	**−**0.02	0.45	(**−**0.11, 0.07)	0.03	0.45	(**−**0.06, 0.11)
**Wheat croplands**													
*Animal-dispersed assemblages*													
ES	—	—	—	—	—	—	—	—	—	—	—	—	—
*Wind-dispersed assemblages*													
ES	287	0.02	0.20	0.07	**−**0.01	0.38	(**−**0.08, 0.06)	**−**0.05	0.73	(**−**0.13, 0.03)	**−**0.01	0.28	(**−**0.04, 0.06)
*Unassisted assemblages*													
ES	290	0.00	0.34	0.22	0.00	0.21	(**−**0.04, 0.04)	**−**0.01	0.28	(**−**0.05, 0.04)	**−**0.00	0.21	(**−**0.04, 0.04)

Full models included three independent variables, resistance distance of woodlands (DistR woodlands), grasslands (DistR grasslands), and croplands (DistR crops), and one dependent variable, the effect size (ES) of similarity values. Models were done for animal-, wind-dispersed and unassisted assemblages for woodland, temporary grassland and wheat cropland habitats. Abbreviation: “−” [a random similarity pattern (ES not different from zero)]. Models were not done in this latter case. Confidence interval that did not encompass zero are in bold.

#### Woodland assemblages

**Figure 3:**
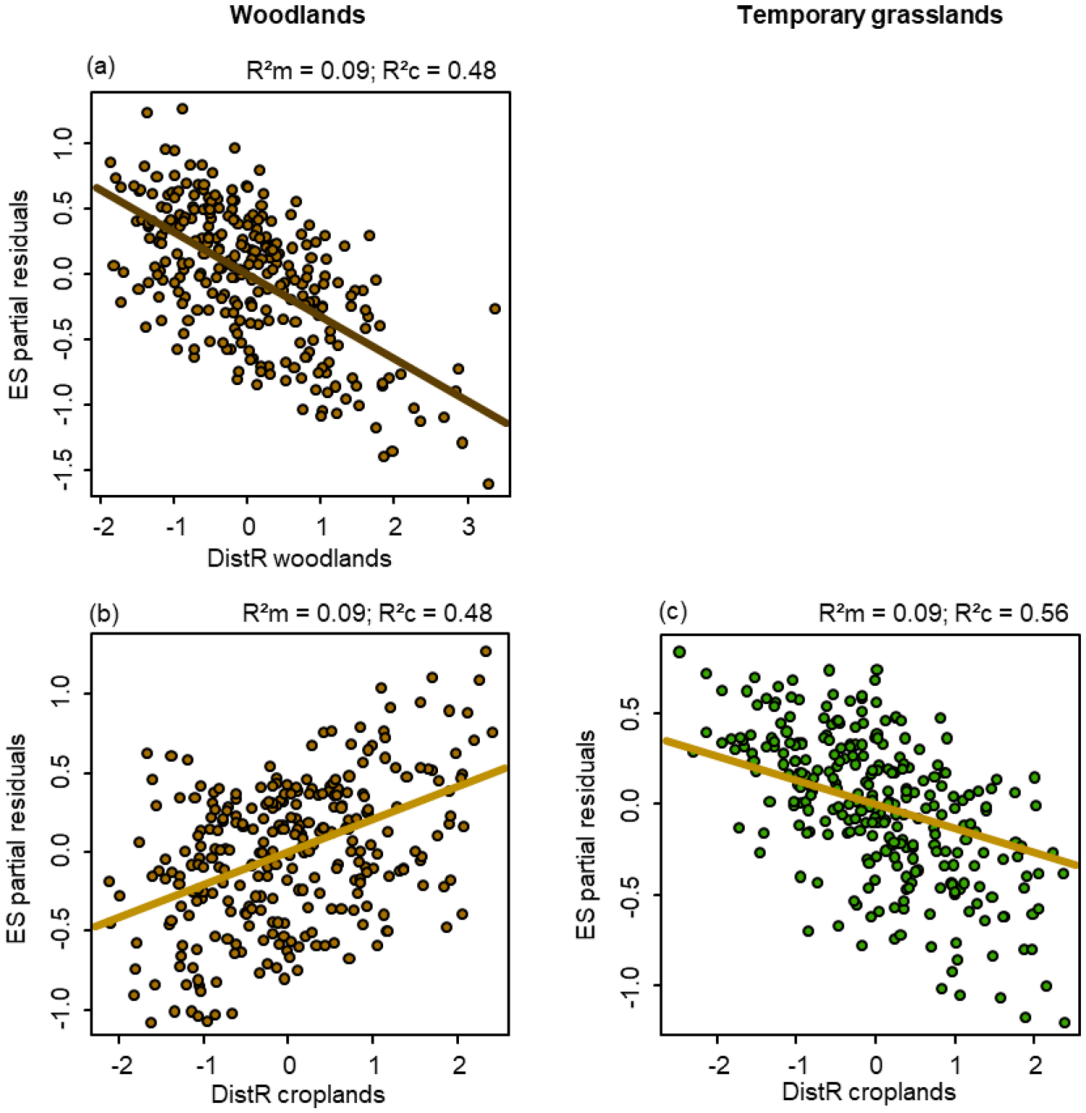
Partial residual plots denoting the significant impact of the resistance distance (distR) of woodlands (brown lines) and croplands (yellow lines) on the effect size (ES) of similarity values for animal-dispersed assemblages in woodlands (left, brown dots) and temporary grasslands (right, green dots). These plots show the effects of a given independent variable when all other independent variables are statistically fixed. The marginal (R²m) and conditional (R²c) R² of the model are on each plot.

Animal-dispersed plant assemblages were more similar than expected when the connectivity provided by woodland habitats increased (dispersal enhancing effect) and when the connectivity provided by cropland habitats decreased (dispersal barrier effect) (Table 2; Fig. 3a,b). The observed similarity values between wind-dispersed and unassisted plant assemblages did not differ from random distributions.

#### Temporary grassland assemblages

Animal-dispersed plant assemblages were more similar than expected by chance when connectivity provided by cropland habitats increased (dispersal enhancing effect) (Fig. 3c), whereas wind-dispersed and unassisted assemblages were independent of connectivity regardless of habitat type on which connectivity was based on (Table 2).

#### Wheat cropland assemblages

Observed similarity values for animal-dispersed assemblages did not differ from random distributions. ES values for wind-dispersed and unassisted assemblages were independent of connectivity (Table 2).

## Discussion

Regardless of habitat type and of the primary dispersal mode, most plant assemblages displayed non-random dispersal patterns, suggesting a dispersal limitation. The three assemblages showing a random dispersal pattern – wind-dispersed and unassisted assemblages of woodlands and animal-dispersed assemblages of wheat croplands – were characterised by low species richness and a restricted regional species pool. These characteristics may explain why observed similarity values were statistically similar to expected similarity values.

Plant assemblages in temporary grasslands and woodlands were dependent on landscape connectivity but wheat cropland assemblages were not. By taking the influences of woodland, grassland and cropland habitats into account simultaneously, we improved our ability to predict species response to changes in connectivity in accordance with our first hypothesis. This suggests that the dispersal of plant species is affected by the connectivity provided by various habitat types simultaneously. As a common pattern for woodland and temporary grasslands, the connectivity provided by grassland habitats had no effect on the similarity of plant assemblages between habitat patches. The absence of any effect has been demonstrated for grassland assemblages^11,12,37,45^ and is extended here to woodland assemblages. Plant dispersal in grassland habitats is mainly achieved by anthropogenic vectors, such as livestock and agricultural machinery^46–48^. Dispersal in grasslands might thus be more dependent on farm logistics than on connectivity. However, the absence of effect here could also be due to our inability to map narrow, linear, herbaceous-dominated habitats, such as grassy strips along the edges of croplands, road and railway verges, even though they are all likely to play an important role in the dispersal of grassland plant species^8,49^. The absence of a landscape connectivity effect in wheat cropland plant assemblages may be explained by two non-exclusive hypotheses: (1) the high proportion (>30%) of croplands in the landscape may be more important to seed dispersal than the role of connectivity, which is in agreement with the fragmentation threshold hypothesis^50^ and (2) the dominance of generalist species (which occupy broad ecological niches) in wheat cropland assemblages, due to the high disturbance rate and environmental variability of cropland habitat. Such generalist species display high dispersal abilities and are not restricted to any specific habitat type^51,52^, which implying that generally, they may not be affected by connectivity^53,54^.

In woodland and temporary grassland assemblages, we detected common patterns of response to the connectivity provided by grassland habitats. However, we did not find any general pattern for the connectivity provided by woodland and cropland habitats. The ability of woodland and cropland habitats to provide connectivity appeared to be dependent on the primary dispersal mode of plant species, in agreement with our second hypothesis.

We partially validated our first sub-hypothesis, since the dispersal of animal-dispersed plants (i.e. similarity between assemblages) was enhanced by the connectivity provided by certain types of habitat. However, their dispersal was also reduced or not affected by others. For example, the dispersal of animal-dispersed plant species inhabiting woodland habitats was enhanced by the connectivity provided by woodland habitats but inhibited by the connectivity provided by cropland habitats. This may be explained by two processes. Movement of animal species is indeed known to preferentially occur through woodland habitats^55^. In contrast, wood-dwelling animal species, such as mammals^56,57^ or birds^58–60^ may avoid open habitats due to the higher risk of predation. We found that in temporary grassland assemblages, dispersal of animal-dispersed plant species was enhanced by the connectivity provided by cropland habitats, but was not affected by other types. We hypothesise that seeds are transferred by animal vectors (i.e. granivorous animal species) from croplands to grasslands during periods of seed dispersal in grasslands. This may occur after cereals have been harvested, when seed resources in croplands are low and animals switch their foraging focus to wild grass seeds. A change from a cropland to a grassland habitat for foraging has already been shown to occur in carabid species^61,62^. Our results suggest an expansion of this concept to the landscape scale.

In contrast to our second sub-hypothesis, we found that wind dispersal was not affected by landscape connectivity even through wind can disperse seeds over long distances^63,64^. Specifically, for these species, we were unable to demonstrate either a dispersal barrier effect of woodland habitats or a dispersal enhancing effect of open habitats (i.e. grassland and cropland habitats), even though such effects have already been demonstrated in simplified experimental conditions^40–42^. One possible explanation for our results is that fine-scale habitat structure may be more important in enhancing or inhibiting seed dispersal than the simple presence or absence of woodland or open habitats. For example, hedgerows vary in height, width, vertical stratification and frequency of gaps, all of which affect small-scale wind patterns and hence, drive wind-dispersal patterns. Similarly, crops vary in height, density and cover depending on the type of management practices applied. Betbeder *et al*.^65^ used new remote sensing techniques to quantify fine-scale landscape structure to improve animal species distribution models at the landscape scale. A similar approach should be used to examine plant distribution. Such studies could be coupled with large-scale wind dynamics data, which could be used to determine the distance, direction, frequency and intensity of seed dispersal patterns^63,64,66,67^.

Unassisted dispersal was independent of landscape connectivity for all assemblage types (temporary grassland and wheat cropland assemblages), thus contradicting our third sub-hypothesis. Despite the fact unassisted species only disperse over a short distance (a minimum of 5 m y^−1^)^68^, these species did not depend on the connectivity provided by their own habitats. Our results suggests that the ploughing up of grassland (every 5 years) and cropland (every 1 year) habitats on average in our study area is probably too frequent for unassisted plant species to disperse from one habitat patch to another. In such intensively managed habitats, connectivity depends on time, which would require incorporating past land-cover maps^11,12^ in the assessment of connectivity effects^69,70^.

Taking into account the additive effects of the connectivity provided by the three types of habitats shed light on the mechanisms of plant dispersal.

We found opposite effects on dispersal of the connectivity provided by habitats of the same type (dispersal enhancing effect) and by the other habitat types (dispersal barrier effect) (e.g. woodland animal-dispersed assemblages). Both enhancement and inhibition may partly compensate for one another. We also found that the connectivity provided by habitat of the same type does not affect plant assemblages of the same habitat, but that provided by other habitat types enhance dispersal (e.g. grassland animal-dispersed assemblages). Such positive effects of other habitat types have been demonstrated at the local scale for animals of a given habitat type [e.g. for birds^71^ and for beetles^72^], leading to the concept of landscape supplementation^73^. Organisms supplement their resource intake by using resources in nearby patches of the same habitat or by using a substitutable resource in nearby patches of a different habitat type^73^. In agreement with Dunning *et al*., our results suggest that plant species could also supplement their “natural” dispersal pathway (i.e habitats of the same type) by using other types of habitats. The supplementation of dispersal pathways for animal-dispersed plant species may be indirectly linked to habitat supplementation by animal species. The validation of this concept in animal-dispersed plant species – and its possible extension to wind-dispersed and unassisted species – is an interesting perspective and could be achieved by analysing the genetic similarity of some representative species of each primary dispersal mode. Using a combination of dispersal pathways would increase a plant’s effective dispersal and improve population fitness, especially in intensified agricultural landscapes where habitats are extremely fragmented.

## Methods

### Study area and selected sites

This study was carried out in the LTSER site “Zone Atelier Armorique” (ZAAr, ca. 13,000 ha) located in Brittany, western France (48° 36′ N, 1° 32′ W). The study area is characterised by a *bocage* landscape dominated by multicrop-livestock farming systems, determined by similar physical constraints. The topology is flat, the soil is on granitic and sandstone bedrocks and the climate is temperate oceanic. The landscape in this study area has been changing since 1955 as a result of successive agricultural policies^74^. Changes included a decrease in the length and the connectivity of the hedgerow network^75^, abandonment of (i) permanent grasslands and (ii) of root and tuber croplands in favour of (i) temporary grasslands (i.e. regularly ploughed) and (ii) maize fields^76^ resulting in an increase in the size of grassland and cropland patches.

We analysed the effect of connectivity on plant assemblages in three habitat types that represent 90% of the landscape: cropland (44%), grassland (31%) and woodland (15%) (for more details about changes over time in the study area, see Supplementary Fig. S1). We assessed the effect of connectivity provided by cropland, grassland and woodland habitats on plant assemblage similarity for each habitat type. For each habitat type studied (wheat croplands, temporary grasslands and woodlands), we selected 25 patches scattered throughout the landscape with a distance of at least 150 m between any two patches to reduce spatial autocorrelation. Patch selection was based on land-cover maps in the ZAAr database. The maps were constructed using aerial imagery based on ten land-cover categories: woodland, grassland, grassy strip, fallow land, maize, cereal cropland, other cropland, built up area, road, and aquatic habitat. The hedgerow network was obtained from the 2016 Kermap (geographic raster) database for woodland habitats. We selected patches of similar size (about 2 ha for woodland, 1 ha for temporary grassland and 2.5 ha for wheat cropland), based on the average patch size of these three habitat types in the study area. We interviewed farmers to determine and standardize patch age, the management regime and previous land cover (i.e. the habitat type on which the patch was established) for woodland, temporary grassland and wheat cropland, and initial species sowing composition for temporary grassland and wheat cropland. Since the initial species composition was not available for planted woodlands, we selected woodland patches with similar dominant tree species composition, assuming they reflect the initial planting composition (Table 3). We checked that patches did not originate from the fragmentation of an initially single habitat patch (up to 1952, which is the oldest land-cover map available in the study area, for woodlands and to previous land cover for temporary grassland and wheat croplands).

**Table 3:** Habitat patches characteristics of woodland, temporary grassland, and wheat cropland habitats.

		Woodlands	Temporary grasslands	Wheat croplands
Size (ha)	Median	2	1	2.5
	Range	1.06–8.00	0.44–2.48	1.95–3.12
Age (years)		>64	6–7	1
Initial sowing species composition			*Trifolium repens* and *Lolium perenne*	*Triticum* sp.
Dominant tree species composition		*Fagus sylvatica*, *Quercus robur* and *Castanea sativa*		
Management regime		Very extensive or absence of management	Grazing with occasional mowing and an occasional use of a soil enrichment product	Intensive crop management
Previous land cover		Cropland or grassland	Cropland	Cropland (*Zea mays*)

### Assessment of similarity among plant assemblages

We performed floristic surveys in the 25 patches of woodland, temporary grassland and wheat cropland selected for the study. Floristic surveys were conducted in six 14 × 5 m quadrats in woodlands, ten 5 × 5 m quadrats in temporary grasslands and five 5 × 5 m quadrats in wheat croplands, i.e. the minimum recommended sampling area for each cover type^77^. These quadrats were located equidistantly from each other in the patch and at least 20 m from the edge of the patch. In each quadrat, we identified species composition and measured species abundance (in percentage cover). Only herbaceous plant species were considered, since ligneous species recorded were related to local management practices linked to tree plantations (i.e. dominant tree species composition of woodlands or dominant tree and shrub species composition of hedgerows surrounding habitat patches). We sampled plant assemblages in woodlands in 2013 (some of these data are already published in Mony *et al*.^24^) and in temporary grasslands and wheat croplands in 2016.

The effect of connectivity on similarity among species assemblages was analysed for each primary dispersal mode. We assessed the primary dispersal mode for each species using the *Baseflor* database^78^ with the following typology: animal-dispersed, wind-dispersed, unassisted (i.e. gravity-dispersed and autochorous species) and water-dispersed species (referred as “other” in Fig. 1, since they were not included in this study). The main animal species in our study area which could be involved in plant dispersal are mostly small species (e.g. insects, small mammals and granivorous birds), following the disappearance of most large wild species due to the low carrying capacities of the remaining natural areas^79^. We focused on the primary dispersal mode for each species, although most species have the ability to be dispersed by more than one vector (2.15 on average^80^), owing to the absence of data on these secondary (or other) dispersal modes in the existing databases. Data on the primary mode of dispersal were available for all the species identified. Plant species in a few genera (e.g. *Carex*, *Epilobium*, *Leotodon, Taxaracum and Oxalis* sp.) could not be determined to species level (5.3% of plants in woodlands, 7.8% in temporary grasslands and 6.4% in wheat croplands). In these particular cases, we assigned the primary dispersal mode of close relatives by assuming phylogenetic conservatism^81^.

For each habitat type, we assessed similarity in plant composition for each pair of habitat patches as a proxy of dispersal^8,23^. Thanks to the standardisation of habitat patches mentioned above, we assumed that similarity in plant composition could substitute the most reliable proxy of dispersal, i.e. genetic similarity, which is restricted to the population level^82^. For each pair of habitat patches, we separately calculated the Sørensen similarity index *S*_*s*ø*r*_ for assemblages of animal-dispersed, wind-dispersed and unassisted species. This index was calculated using the ade4 package^83^. The higher the similarity index (*S*_*s*ør_), the higher the similarity between a given pair of habitat patches.

### Assessment of landscape connectivity between habitat patches

**Figure 4:**
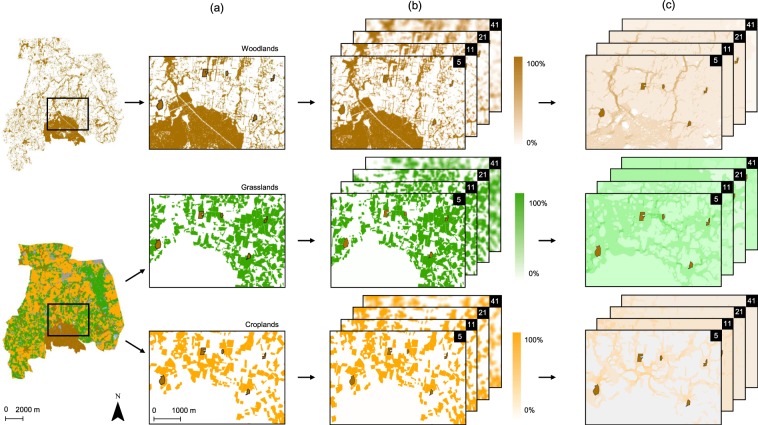
Illustration of the method used to model connectivity as provided by woodland, grassland and cropland habitats. Example of sampled woodland habitats. Sampled woodlands are indicated in brown and surrounded with a black edge on all maps. (**a**) Extraction of woodland, (top), grassland (middle) and cropland (bottom) habitats derived from (i) the Kermap geographic raster database for woodland habitats and (ii) the land-cover map of the study area. (**b**) Calculation of the proportion of woodland, grassland and cropland habitat derived from the number of pixels in circular sliding windows of 5-, 11-, 21- and 41-diameter pixels. (**c**) Electric current fluxes based on circuit theory between woodlands, based on woodland, grassland and cropland habitats. For a given habitat type, a focal pixel for which the proportion of the habitat over the window (i.e. focal neighbourhood) is 100% was assigned a resistance value of 1 (i.e. highly suitable to dispersal), whereas a focal pixel for which the habitat proportion is 0% was assigned a resistance value of 100 (i.e. slightly suitable to dispersal).

For each habitat type (see Fig. 4 for the example of sampled woodland habitats), we assessed the landscape connectivity provided by three types of habitat: woodland, grassland and cropland. We considered woodland habitats as forests, woodlots, and hedgerows; grassland habitats as grasslands, grassy strips and fallow lands and cropland habitats as maize, cereal cropland and other cropland types and grouped these land-cover types accordingly based on our land-cover maps (Fig. 4a). The landscape connectivity provided by each habitat type (woodland, grassland and cropland) was measured for all pairs of habitat patches in each habitat type (i.e. 25 habitat patches is equivalent to 300 pairs of habitat patches for each habitat type).

We used circuit theory^21,22^ to model connectivity between patches. This modelling method depends on random walk theory and incorporates the contributions of all dispersal pathways to evaluate the degree of connectivity between patches. These contributions depend on the resistance to movement between habitats. The connectivity value that results from this modelling method is a measure of the degree of isolation, called resistance distance: the higher the resistance distance, the lower the connectivity.

### Creating resistance maps

This modelling method requires the creation of resistance maps. Resistance maps were created using sliding (moving) window analysis, which makes it possible to assign a resistance value to each focal pixel of the landscape, the value being calculated and mediated by the neighbouring pixel values^84^. Based on the gradient (or continuum) model^84,85^, this method avoids assigning resistance values based on a discrete representation of the landscape matrix^84–88^. For each habitat type, resistance values were calculated as one hundred and one minus the proportion of the habitat type in a given landscape window, here simulated as a sliding circular window of a given diameter. A focal pixel for which the proportion of the habitat type over the window (i.e. focal neighbourhood) is 100% was assigned a resistance value of 1 (i.e. highly suitable to dispersal). Conversely, a focal pixel for which the proportion of the habitat type is 0% was assigned a resistance value of 101 (i.e. slightly suitable to dispersal) (see Supplementary Fig. S2). The creation of resistance maps based on the proportion of woodland, grassland and cropland habitat for each habitat type reduced subjectivity (i.e. expert knowledge) and avoided hierarchisation of habitat types when assigning resistance values. The proportion of woodland, grassland and cropland habitats was calculated from the number of pixels in the circular sliding window with a displacement and interpolation values of one pixel (i.e. 5 m). We tested sliding windows of different diameters of 5 (25 m), 11 (55 m), 21 (105 m) and 41 (205 m) pixels – closely linked to the range of local dispersal distances of plants^41,89^ – to test the sensitivity of the connectivity indices to continuous landscape representation (Fig. 4b). These resistance maps were produced using Chloe2012 software^90^.

### Landscape connectivity assessment

We calculated landscape connectivity using resistance distance. For each pair of habitat patches, we calculated the resistance distance based on the resistance maps using Circuitscape software^22^ (Fig. 4c). Graphab 3.0.2 software^91^ was used to generate Euclidean links between each pair of habitat patches for each type of habitat. Pairs of habitat patches located at the boundary of the study area, for which Euclidean links were outside the study area, were excluded from subsequent analyses. We used Pearson correlations to examine relationships among the different resistance distances we calculated for the various diameters (5–205 m) of the sliding windows. Resistance distances obtained from habitats of the same habitat type for the different sliding window sizes were strongly correlated (*r* ≥ 0.95, p < 0.001). We then selected resistance distances obtained from the intermediate-diameter sliding window (11 pixels, i.e. 55 m).

## Statistical analysis

### Detection of non-random response patterns using a null model

To focus on non-stochastic processes, we assessed the effect of connectivity on the similarity between pairs of assemblages (observed *S*_*s*ør_ similarity) according to the distribution of the similarity index based on the calculation of random assemblages (expected *S*_*s*ør_ similarity) using a null model. This random similarity reflected random dispersal of seeds (i.e. a lack of a dispersal filter). The null distribution was calculated from data on assemblages derived from a random sampling of species from the regional species pool^92^. Random assemblages had similar species richness to the corresponding observed assemblage. The probability of each species being selected in the random assemblages was weighted by the occurrence of the species within the regional species pool considered (i.e. all recorded observed species). This process was repeated for each primary dispersal mode and for each type of habitat.

An effect size (ES) was calculated based on the probability that the observed value *S*_*s*ør_ was lower than the value expected under the null hypothesis (i.e. the quantile of the null distribution for which the observed value was derived^93,94^, see Supplementary Methods for calculation). ES of similarity values vary between –1 and 1. In our study, when the ES was close to zero, the observed similarity value between each pair of assemblages was considered to be random. Due to the non-normality of ES values, we used a Wilcoxon test, which is routinely used to test whether ES values were significantly overall different from zero^95–98^, and thus for the significance of non-random plant assemblage similarities between pairs of habitat patches. When the Wilcoxon test was significant, negative ES values indicated that observed similarity values between each pair of assemblages were lower than expected by chance under the null hypothesis (i.e. dispersal barrier effect). In contrast, positive ES values revealed that the observed similarity values were higher than expected under the null hypothesis (i.e. dispersal enhancing effect).

### Influence of landscape connectivity on similarity between plant assemblages

We used linear mixed models to assess the influence of landscape connectivity on the similarity of plant assemblages using the ES of similarity value for each pair of assemblages and primary dispersal mode as the dependent variable and the resistance distances obtained between each pair of habitat patches as independent variables for: (1) woodland, (2) grassland and (3) cropland. To account for the partial dependence of the data on matrices of ES values, the two habitat patches comprising each pair of assemblages analysed were considered as the two random effects^23,99,100^. In order to ensure unbiased (i) estimates of fixed effects and (ii) model selection procedures^101–106^, these linear mixed models were fitted using the maximum likelihood (ML) estimation of the variance components. Resistance distances were centered and scaled to ensure the regression coefficients were comparable among models. Each model was performed using a model-averaging method, which takes model selection uncertainty into account^107^. We built all possible models based on all additive combinations of explanatory variables and ranked them using the Akaike information criterion corrected for small sample sizes (AICc). We then generated full-model averaged estimates of each independent variable across the most parsimonious models^107^ (ΔAICc < 4, Supplementary Table S1). We considered independent variables to be significant if their 95% confidence intervals did not overlap with zero^107^. Finally, we calculated the proportion of variance explained by fixed effects (marginal R²) and by fixed and random effects (conditional R²) of the most parsimonious model^108^.

We also checked whether the effect of connectivity on plant assemblage similarity was not indirectly due to the patches having similar local environmental conditions. To this end, we used the similarity of Ellenberg’s indicator values between each pair of assemblages [moisture, nutrient availability and pH], in addition to using resistance distances to explain ES similarity values (see Supplementary Methods). The results of the averaged models obtained from these preliminary analyses were consistent with the averaged models, which included only the resistances distances as fixed effects (Supplementary Table S2). This confirmed that the relationships we demonstrated between resistance distance and plant assemblage similarity were robust.

All statistical analyses were performed using R.3.5.1 (R Core Team). Linear mixed models, model averaging and associated marginal and conditional R² were calculated using lmerTest^109^, MuMIn^110^ and piecewiseSEM^111^ packages.

## Supplementary information

Supplementary Information

## Data Availability

All data and scripts used to perform the main analyses are available on Figshare (10.6084/m9.figshare.7321268).
