# Supplementary material for: Additive effects of connectivity provided by different habitat types drive plant assembly
Source: Sci Rep. 2019 Sep 27;9:13952. doi: 10.1038/s41598-019-50184-2 (PMC6764998; doi:10.1038/s41598-019-50184-2)
Supplement: Supplementary file 1 — Supplementary Information [file 41598_2019_50184_MOESM1_ESM.pdf]

Supplementary Information.

Léa Uroy, Cendrine Mony and Aude Ernoult

Additive effects of connectivity provided by different habitat types drive plant assembly.

Scientific Reports.

Table of contents:

Figure S1. 2

Figure S2. 3

Supplementary Methods 4

Detection of non-random response patterns using a null model 4

Influence of landscape connectivity on similarity between plant assemblages 4

Table S1 6

Table S2 9

References 11

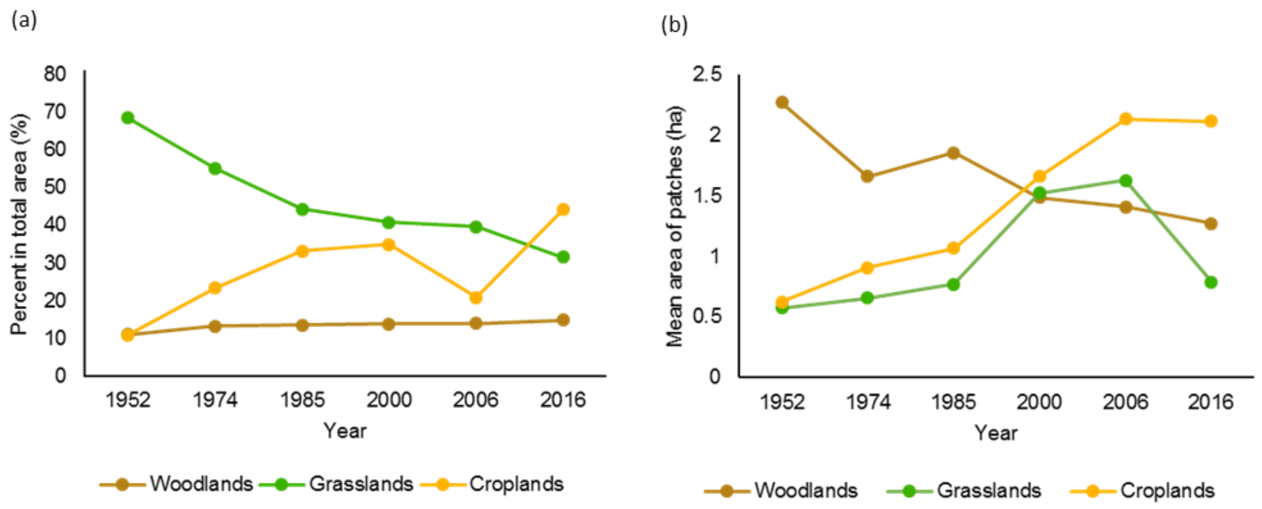

Figure S1. (a) Percentage of total area and (b) mean area of patches in the study area over time (1952 – 2016) for each habitat type. These metrics have been measured from land-cover maps in the ZAAr database.

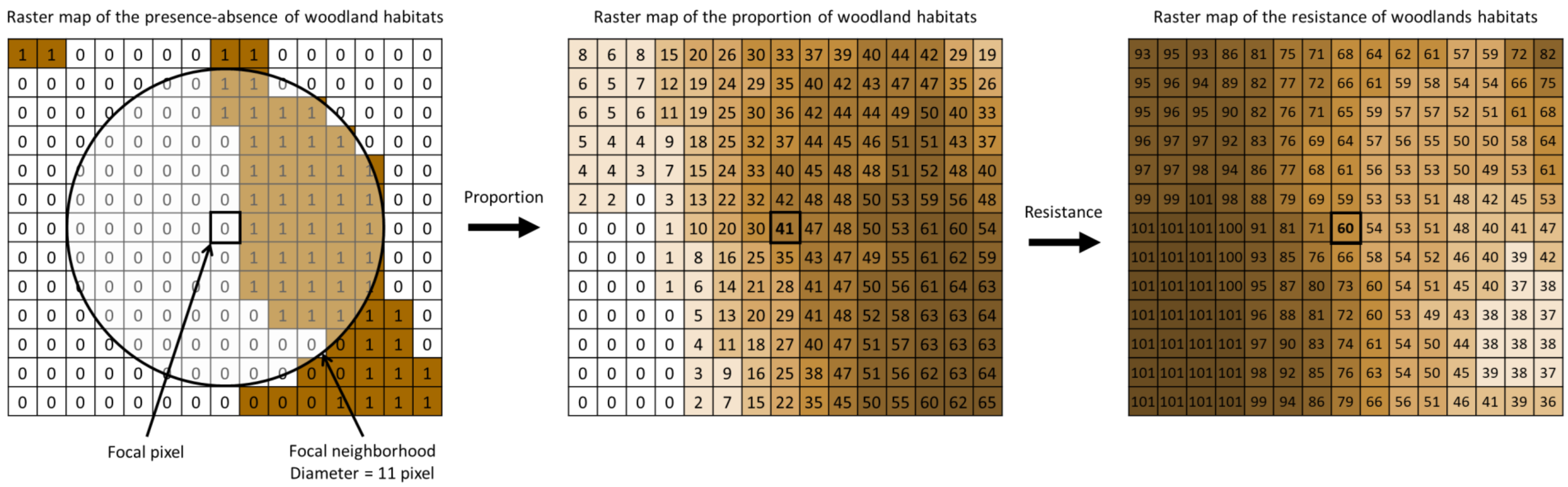

Figure S2. Schematic representation of the sliding (moving) circular window analysis used to create resistance maps for each habitat type. Example of woodlands habitats in a sliding circular window of 11 pixels (55 meters). Resistance values were calculated as one hundred and one minus the proportion of woodland habitats in a given landscape window, here simulated as a sliding circular window of a given diameter. A focal pixel for which the proportion of the habitat type over the window (i.e. focal neighbourhood) is 100% was assigned a resistance value of 1 (i.e. highly suitable to dispersal). Conversely, a focal pixel for which the proportion of the habitat type is 0% was assigned a resistance value of 101 (i.e. slightly suitable to dispersal).

Supplementary Methods

Detection of non-random response patterns using a null model

Each observed value $S_{s\o\mathrm{r}}$ was compared to its null distribution by calculating the one-tailed probability P that the observed value $S_{s\o\mathrm{r}}$ was lower than the value expected under the null hypothesis. That is, the quantile of the null distribution for which the observed value was derived as follows [1,2]: $P = \frac{\sum S_{s\o\mathrm{r}}(null<obs)+\sum\frac{S_{s\o\mathrm{r}}(null=obs)}{2}}{1000}$, where $S_{s\o\mathrm{r}}(null < obs)$ = number of times in which the expected value $S_{s\o\mathrm{r}}$ was lower than the observed value and $S_{s\o\mathrm{r}}(null = obs)$ = number of times in which the expected value $S_{s\o\mathrm{r}}$ was equal to the observed value. This one-tailed probability was then used to compute an effect size (ES) of similarity as follows: $ES = (P - 0.5) \times 2$ [1,2]. This calculation of effect size was preferred to the Standardized Effect Size (SES) due to the asymmetry and non-Gaussian shape of the majority of null distributions we found with our data[3].

Influence of landscape connectivity on similarity between plant assemblages

We checked whether the effect of landscape connectivity on plant assemblage similarity was not indirectly due to the patches having similar local environmental conditions. The similarity of Ellenberg's indicator values $S_{E_{ij}}$ between each pair of assemblages [moisture, nutrient availability and pH, extracted from the *Baseflor* database] was used as a proxy of the similarity of environmental conditions. These similarities of Ellenberg's indicator values were calculated as $S_{E_{ij}} = |\bar{E}_i - \bar{E}_j|$, where $\bar{E}_i$ and $\bar{E}_j$ are the mean of Ellenberg's indicator values of species recorded in the *i* and *j* assemblages from habitat patches. We thus tested the effect of both i) resistance distance between pairs of habitat patches and ii) similarity of Ellenberg's indicator values for the same pairs on ES similarity values. These relationships were investigated using linear mixed models, using both resistance distances and the similarity of Ellenberg's indicator values for the same pairs as a fixed effect and the two habitat patches constituting the pair of

assemblages as the two random effects. We then performed a model-averaging method following the same procedure as the main analyses of the manuscript.

Table S1. Summary of the most parsimonious regression models according to the AICc framework used for model averaging. Full models included three independent variables, resistance distance of woodlands (DistR woodlands), grasslands (DistR grasslands), and croplands (DistR crops), and one dependent variable, the effect size (ES) of similarity values. Models were done for animal-, wind-dispersed and unassisted assemblages for woodland, temporary grassland and wheat cropland habitats. Abbreviations: “x” [the independent variable(s) included in non-significant models] and “-" [a random similarity pattern (ES not different from zero)]. Models were not done in this latter case. The significance of each model was assessed by comparing the defined and null models [i.e. model without any independent variable(s)] using a likelihood ratio test[4,5]. The significance of each independent variable in each model was assessed with ANOVA, based on type II sums of squares. Models in bold are significant. ***: $p < 0.001$. **: $p < 0.01$; *: $p < 0.05$; ns: $p > 0.05$.

	Models					Intercept	DistR woodlands			DistR grasslands			DistR croplands		
	N	AICc	R^2m	R^2c	p		Estimate	χ^2	p	Estimate	χ^2	p	Estimate	χ^2	p
**Woodlands**															
Animal-dispersed assemblages															
ES	283	425.0	0.09	0.463	***	0.20	-0.26	16.74	***				0.20	8.68	**
	283	425.5	0.10	0.503	***	0.21	-0.39	11.40	***	0.13	1.77	ns	0.21	9.29	**
Wind-dispersed assemblages															
ES	-	-	-	-	-	-	-	-	-	-	-	-	-	-	-
Unassisted assemblages															
ES	-	-	-	-	-	-	-	-	-	-	-	-	-	-	-
**Temporary grasslands**															
Animal-dispersed assemblages															
ES	283	222.2	0.09	0.56	**	0.62				0.05	2.67	ns	-0.14	11.53	***
	283	222.9	0.05	0.54	**	0.62							-0.10	9.38	**
	283	222.3	0.08	0.56	**	0.62	0.05	1.78	ns				-0.14	9.89	**
	283	224.5	0.09	0.57	**	0.62	-0.00	0.00	ns	0.05	0.86	ns	-0.14	10.28	**
Wind-dispersed assemblages															
ES	283	226.5	0.00	0.62	ns										
	283	227.0	0.00	0.63	ns					x	x	x			
	283	227.3	0.00	0.62	ns		x	x	x						
	283	228.1	0.00	0.63	ns								x	x	x
	283	229.0	0.01	0.63	ns					x	x	x	x	x	x
	283	229.1	0.00	0.63	ns		x	x	x	x	x	x			
	283	229.4	0.01	0.62	ns		x	x	x				x	x	x
Unassisted assemblages															
ES	283	278.1	0.04	0.45	**	0.59	-0.09	8.00	**						
	283	278.3	0.06	0.46	**	0.60	-0.12	9.53	**				0.06	1.93	ns
	283	279.3	0.02	0.44	**	0.59				-0.07	6.77	**			
	283	280.2	0.03	0.45	*	0.59	-0.08	1.18	ns	-0.01	0.03	ns			
	283	280.3	0.04	0.45	*	0.59				-0.09	7.31	**	0.04	1.02	ns
	283	280.4	0.06	0.46	*	0.59	-0.11	2.12	ns	-0.02	0.07	ns	0.06	1.97	ns

**Wheat croplands**															
Animal-dispersed assemblages															
ES	-	-	-	-	-	-	-	-	-	-	-	-	-	-	-
Wind-dispersed assemblages															
ES	**287**	**289.9**	**0.02**	**0.20**	*****	**0.07**				**-0.06**	**5.12**	*****			
	287	291.5	0.03	0.20	ns					x	x	x	x	x	x
	287	291.9	0.02	0.20	ns		x	x	x	x	x	x			
	287	292.0	0.01	0.19	ns		x	x	x						
	287	292.8	0.00	0.17	ns										
	287	293.4	0.03	0.20	ns										
	287	293.5	0.02	0.18	ns		x	x	x				x	x	x
Unassisted assemblages															
ES	290	388.7	0.00	0.34	ns										
	290	390.6	0.00	0.34	ns					x	x	x			
	290	390.7	0.00	0.34	ns								x	x	x
	290	390.7	0.00	0.34	ns		x	x	x	x	x	x			
	290	392.5	0.00	0.34	ns										
	290	392.6	0.00	0.34	ns								x	x	x

Table S2. Summary of the model-averaged estimates, relative importance (RI) and 95% confidence interval (CI) of the independent variable(s) according to the AICc framework. Full models included six independent variables, resistance distance of woodlands (DistR woodlands), grasslands (DistR grasslands), and croplands (DistR crops) and similarity edaphic indicator values of nutrient avaibility ($S_{E_{ij}}$ Nutrient), moisture ($S_{E_{ij}}$ Moisture) and pH ($S_{E_{ij}}$ pH) and one dependent variable, the effect size (ES) of similarity values. Negative ES values indicate a lower similarity value than expected, due to a dispersal (if a significant effect of one or several distR variables is detected) and/or local environmental conditions (if a significant effect of one or several variables $S_{E_{ij}}$ is detected) effect(s). Positive ES values indicate a higher similarity than expected, due to a dispersal (if a significant effect of one or several distR variables is detected) and/or local environmental conditions (if a significant effect of one or several variables $S_{E_{ij}}$ is detected) effect(s). Models were done for animal-, wind-dispersed and unassisted assemblages for woodland, temporary grassland and wheat cropland habitats. Abbreviations: “-" [a random similarity pattern (ES not different from zero)]. Models were not done in this latter case. Confidence interval that did not encompass zero are in bold.

	Models			Intercept	DistR woodlands			DistR grasslands			DistR crops			$S_{E_{ij}}$ Nutrient			$S_{E_{ij}}$ Moisture			$S_{E_{ij}}$ pH		
	N	R²m	R²c		Estimate	RI	CI	Estimate	RI	CI	Estimate	RI	CI	Estimate	RI	CI	Estimate	RI	CI	Estimate	RI	CI
**Woodlands**																						
Animal-dispersed assemblages																						
ES	283	0.23	0.55	0.19	**-0.30**	**1.00**	**(-0.51, -0.08)**	0.06	0.49	(-0.11, 0.24)	**0.16**	**1.00**	**(0.04, 0.29)**	-0.01	0.31	(-0.07, 0.05)	**-0.15**	**1.00**	**(-0.21, 0.09)**	**-0.17**	**1.00**	**(-0.25, -0.10)**
Wind-dispersed assemblages																						
ES	-	-	-	-	-	-	-	-	-	-	-	-	-	-	-	-	-	-	-	-	-	-
Unassisted assemblages																						
ES	-	-	-	-	-	-	-	-	-	-	-	-	-	-	-	-	-	-	-	-	-	-
**Temporary grasslands**																						
Animal-dispersed assemblages																						
ES	283	0.08	0.58	0.63	0.02	0.37	(-0.06,0.09)	0.02	0.38	(-0.05, 0.08)	**-0.10**	**0.97**	**(-0.20,-0.01)**	0.01	0.29	(-0.03, 0.04)	-0.00	0.24	(-0.03,0.02)	**-0.09**	**1.00**	**(-0.14, -0.05)**
Wind-dispersed assemblages																						
ES	283	0.00	0.62	0.50	-0.01	0.27	(-0.06, 0.04)	-0.01	0.35	(-0.06, 0.04)	-0.00	0.20	(-0.04, 0.04)	-0.01	0.35	(-0.05, 0.03)	-0.01	0.27	(-0.04, 0.03)	-0.00	0.19	(-0.02, 0.02)
Unassisted assemblages																						
ES	283	0.0	0.45	0.59	-0.08	0.77	(-0.20, 0.04)	-0.02	0.38	(-0.10, 0.06)	0.03	0.47	(-0.06, 0.12)	-0.01	0.31	(-0.05, 0.03)	-0.01	0.32	(-0.05, 0.03)	0.00	0.17	(-0.02, 0.02)
**Wheat croplands**																						
Animal-dispersed assemblages																						
ES	-	-	-	-	-	-	-	-	-	-	-	-	-	-	-	-	-	-	-	-	-	-
Wind-dispersed assemblages																						
ES	287	0.07	0.22	0.07	-0.01	0.32	(-0.06, 0.05)	-0.06	0.88	(-0.14, 0.02)	0.01	0.25	(-0.04, 0.05)	0.05	0.75	(-0.03, 0.12)	-0.03	0.61	(-0.09, 0.03)	-0.07	1.00	(-0.12, -0.02)
Unassisted assemblages																						
ES	290	0.04	0.33	0.22	-0.00	0.15	(-0.03, 0.03)	-0.01	0.24	(-0.05, 0.04)	-0.00	0.15	(-0.03, 0.03)	0.04	0.63	(-0.05, 0.13)	-0.05	0.75	(-0.14, 0.03)	0.00	0.14	(-0.03, 0.03)

References

1. Kelt, D. A., Taper, M. L. & Meserve, P. L. Assessing the Impact of Competition on Community Assembly: A Case Study using Small Mammals. *Ecology* **76**, 1283–1296 (1995).
2. Chase, J. M., Kraft, N. J. B., Smith, K. G., Vellend, M. & Inouye, B. D. Using null models to disentangle variation in community dissimilarity from variation in α-diversity. *Ecosphere* **2**, 1–11 (2011).
3. Bernard-Verdier, M. *et al.* Community assembly along a soil depth gradient: contrasting patterns of plant trait convergence and divergence in a Mediterranean rangeland. *J Ecol* **100**, 1422–1433 (2012).
4. Pinheiro, J. & Bates, D. *Mixed-Effects Models in S and S-PLUS*. (Springer-Verlag, 2000).
5. Bolker, B. M. *et al.* Generalized linear mixed models: a practical guide for ecology and evolution. *Trends Ecol. Evol. (Amst.)* **24**, 127–135 (2009).
